# HPV Genotypes in High Grade Cervical Lesions and Invasive Cervical Carcinoma as Detected by Two Commercial DNA Assays, North Carolina, 2001–2006

**DOI:** 10.1371/journal.pone.0034044

**Published:** 2012-03-29

**Authors:** Susan Hariri, Martin Steinau, Allen Rinas, Julia W. Gargano, Christina Ludema, Elizabeth R. Unger, Alicia L. Carter, Kathy L. Grant, Melanie Bamberg, James E. McDermott, Lauri E. Markowitz, Noel T. Brewer, Jennifer S. Smith

**Affiliations:** 1 Division of STD Prevention, National Center for HIV/AIDS, Viral Hepatitis, STD, and TB Prevention, Centers for Disease Control and Prevention, Atlanta, Georgia, United States of America; 2 Division of High-Consequence Pathogens and Pathology, National Center for Emerging and Zoonotic Infectious Diseases, Centers for Disease Control and Prevention, Atlanta, Georgia, United States of America; 3 Gillings School of Global Public Health and Lineberger Comprehensive Cancer Center, University of North Carolina, Chapel Hill, North Carolina, United States of America; 4 Epidemic Intelligence Service, Centers for Disease Control and Prevention, Atlanta, Georgia, United States of America; 5 Laboratory Corporation of America® Holdings, Burlington, North Carolina, United States of America; 6 Duke University Health System, Durham, North Carolina, United States of America; 7 Carolinas Medical Center, Charlotte, North Carolina, United States of America; Instituto de Higiene e Medicina Tropical, Portugal

## Abstract

**Background:**

HPV typing using formalin fixed paraffin embedded (FFPE) cervical tissue is used to evaluate HPV vaccine impact, but DNA yield and quality in FFPE specimens can negatively affect test results. This study aimed to evaluate 2 commercial assays for HPV detection and typing using FFPE cervical specimens.

**Methods:**

Four large North Carolina pathology laboratories provided FFPE specimens from 299 women ages18 and older diagnosed with cervical disease from 2001 to 2006. For each woman, one diagnostic block was selected and unstained serial sections were prepared for DNA typing. Extracts from samples with residual lesion were used to detect and type HPV using parallel and serial testing algorithms with the Linear Array and LiPA HPV genotyping assays.

**Findings:**

LA and LiPA concordance was 0.61 for detecting any high-risk (HR) and 0.20 for detecting any low-risk (LR) types, with significant differences in marginal proportions for HPV16, 51, 52, and any HR types. Discordant results were most often LiPA-positive, LA-negative. The parallel algorithm yielded the highest prevalence of any HPV type (95.7%). HR type prevalence was similar using parallel (93.1%) and serial (92.1%) approaches. HPV16, 33, and 52 prevalence was slightly lower using the serial algorithm, but the median number of HR types per woman (1) did not differ by algorithm. Using the serial algorithm, HPV DNA was detected in >85% of invasive and >95% of pre-invasive lesions. The most common type was HPV16, followed by 52, 18, 31, 33, and 35; HPV16/18 was detected in 56.5% of specimens. Multiple HPV types were more common in lower grade lesions.

**Conclusions:**

We developed an efficient algorithm for testing and reporting results of two commercial assays for HPV detection and typing in FFPE specimens, and describe HPV type distribution in pre-invasive and invasive cervical lesions in a state-based sample prior to HPV vaccine introduction.

## Introduction

Reducing the burden of cervical cancer is the key benefit of HPV prophylactic vaccines. In the U.S., state-based cancer registries will serve as the basis for evaluating vaccine impact on invasive HPV-associated cancers, including cervical cancers, as these lesions are reportable [1]. However, given the long time period between type-specific HPV infection and development of invasive cancer, this impact will not be detectable for decades [2]. Pre-invasive cervical lesions detected through screening can serve as intermediate measures of vaccine impact in settings where routine cervical cancer screening and systematic reporting systems are established [3]. Although not all state cancer registries currently require reporting of preinvasive cervical lesions (cervical intraepithelial neoplasia grades 2 and 3 [CIN 2,3], adenocarinoma-in-situ [AIS]), sentinel systems have been established across the U.S. to monitor the population impact of HPV vaccine on these clinical endpoints [4].

In addition to monitoring pre-invasive and invasive cervical endpoints, determining HPV type-distribution in these lesions is important to ensure vaccine-associated types are decreasing as vaccination coverage increases. Linking state cancer registries and sentinel systems to diagnostic pathology laboratories makes it possible to retrieve representative tissue for HPV detection and typing. Standardized laboratory methods adapted for optimal HPV testing in these archived formalin-fixed paraffin-embedded (FFPE) tissues are needed to assure that time trends in data reflect reliable measurement [5]. It is recognized that the yield and quality of DNA from FFPE tissue is limited, and even with optimal methods of extraction, the size limit for reliable amplification from FFPE is around 450 base pairs (bp). The PGMY09/11 primers, used in the Roche Linear Array HPV Genotyping Assay (LA), generate a 450 bp amplicon, raising concerns that this method may not yield optimal results in FFPE. An alternative is the SPF primer system used in the Innogenetics Line Probe Assay (LiPA), which generates a 65 bp amplicon. Wheeler et al. [6] used LA and FFPE samples, with re-testing of negative or inadequate samples with a non-commercial version of LiPA. This is an attractive option, but the impact of using both assays in this sequential manner on the observed type-distribution has not been formally investigated.

The objectives of this study were to 1) develop an efficient algorithm for testing and reporting results of two commercial assays for HPV detection and typing in archived FFPE tissue specimens (LA and LiPA), and 2) describe HPV type distribution in CIN2/3, AIS, squamous cell carcinoma (SCC), and adenocarcinoma (ADC) in a state-based sample prior to HPV vaccine introduction.

## Materials and Methods

This study was approved by the University of North Carolina at Chapel Hill Institutional Review Board. Written consent from the patients was waived by the ethics board because the data were obtained and analyzed anonymously.

### Study Samples

Four pathology laboratories in North Carolina participated in this study: Carolinas Medical Center, Duke University Health System, Laboratory Corporation of America® Holdings, and the University of North Carolina Health Care. Combined, these laboratories serve a large majority of the population in North Carolina and cover a wide geographical distribution within the state. Each lab searched their pathology database to generate a complete list of CIN2, CIN3, AIS, and invasive cervical cancer cases diagnosed in women ages 18 years and older from January 1, 2001 to June 1, 2006. After stratifying cases by diagnosis (i.e., CIN2, CIN3/AIS, and invasive cervical cancer), a random sample of 25 cases was selected within each stratum (∼300 samples in total). One block representative of the histology from each of the selected cases was retrieved for type-specific HPV DNA testing. Demographic information retrieved from the pathology records that was linked to the de-identified case number included histologic diagnosis (CIN2, CIN3, AIS, SCC, ADC, adenosquamous cell carcinoma, and other invasive cervical cancer), and other data when available, including age at diagnosis, race, and insurance coverage.

### Laboratory Procedures

**Histopathology review:** Serial sections were cut from each block using precautions to prevent PCR contamination between cases, including single-use disposable microtome blades, cleaning the microtome between cases, and direct transfer of sections for PCR from microtome to sterile tubes using new single-use applicators (no contact with waterbath). The first and last sections were stained with Hematoxylin and Eosin (H&E). Intervening sections were transferred into 2 mL conical screw cap tubes with tether caps, one 10-micron section or two 5-micron sections per tube (Simport, Beloeil, Canada). A study pathologist (ERU) reviewed the H&E stained sections to confirm that histology was representative of the diagnosis. Samples that did not have representative material were not processed.

**DNA isolation:** DNA was extracted and purified by a modified protocol using the DNeasy kit (Qiagen, Valencia, CA) as described elsewhere [7]. Briefly, sections were heated for 20 min at 120°C in 180 µl ATL lysis buffer, then incubated with Proteinase K overnight at 65°C and further purified with spin columns. DNA was eluted in a final volume of 100 µL and tested immediately or stored at −20°C. For every batch of 28 samples, a water blank was processed through all steps of extraction to serve as a “contamination control”.

**HPV Genotyping Tests:** All DNA extracts were tested with LA and LiPA HPV typing assays, both based on L1 consensus PCR and type-specific hybridization. The LA (Roche Diagnostics, Indianapolis, IN) uses biotinylated PGMY09/11 and β-globin primer sets and detects 37 individual HPV types (6, 11, 16, 18, 26, 31, 33, 35, 39, 40, 42, 45, 51, XR(52), 53, 54, 55, 56, 58, 59, 61, 62, 64, 66, 67, 68, 69, 70, 71, 72, 73, 81, 82, 83, 84, 89, IS39) along with β-globin as an internal control. The manufacturer's protocol was followed with the exceptions of using 10 µL extract in the 100-µL PCR reaction and automated hybridization and washing of the reverse line blot with the Beeblot instrument (Bee Robotics, Caernarfon, UK). Samples positive for the XR(52) probe on the LA HPV strip that were also positive for HPV33, 35 and 58 were further evaluated to confirm or exclude the presence of HPV 52 using an HPV 52 quantitative PCR assay with a threshold of 50 viral copies [8]. Samples negative for both β-globin and HPV were considered inadequate for evaluation.

LiPA (Innogenetics, Gent, Belgium) uses SPF10 primers and detects 28 HPV types (6, 11, 16, 18, 26, 31, 33, 35, 39, 40, 43, 44, 45, 51, 52, 53, 54, 56, 58, 59, 66, 68, 69, 70, 71, 73, 74, 81, 82) as well as a generic probe to detect other HPV types (HPV X) and a genomic control probe. The PCR and amplicon hybridization to the HPV genotyping strip (AutoBlot 3000H, MedTec, Buffalo, IL) followed the manufacturer's protocols. The manufacturer's algorithm for interpretation of typing result based on pattern of positive probes was followed. This algorithm includes reporting unequivocal detection of types as well as “possible types”. The ambiguity results from certain hybridization patterns and affects only types 39, 52, 54, 68, 69, and 71. Samples negative for both the genomic control probe and HPV were considered inadequate for evaluation.

### Statistical Analysis

**Concordance between LA and LiPA:** For specimens with adequate LA and LiPA results, we calculated kappa to assess congruence of the type-specific results and performed McNemar's test to identify differences in the marginal frequencies by typing assay. Because of the large number of comparisons, we used a threshold of p<.01 to assess significance of the McNemar's test. We performed these analyses for each HPV type present in both tests. We defined HPV 16, 18, 31, 33, 35, 39, 45, 51, 52, 56, 58, 59, 66, and 68, as high risk (HR) HPV types, HPV 26, 53, 67, 69, 70, 73, 82, 85, and IS39 as “Possible HR”, and HPV 6, 11, 32, 40, 42, 43, 44, 54, 55, 61, 62, 64, 71, 72, 74, 81, 83, 84, 87, 89 as low risk (LR) HPV [9], [10]. Concordance evaluations were restricted to unequivocal positive LiPA results and repeated with “possible type” LiPA results interpreted as positive.

We evaluated two algorithms for combining the LA and LiPA results. These were 1) *parallel algorithm*, in which results of both tests were considered for all samples (positive result = positive for either test) and 2) *serial algorithm*, in which the LiPA assay results were only considered for those samples that were inadequate or negative for any HPV type by LA. We calculated the type-specific prevalence and overall HR or LR prevalence by each algorithm. We also calculated the mean and median number of HPV types detected per woman for each algorithm.

**HPV Type Distribution:** We examined HPV type distribution stratified by grade of cervical diagnosis. For evaluating vaccine-type HPV prevalence, we examined cases positive for HPV16 or 18 within each diagnosis stratum. We used Pearson's χ^2^ test for independence to evaluate bivariate associations between vaccine-type HPV prevalence and race, stratified by diagnosis category. The Cochran-Armitage test was used to examine age trends of HPV 16/18 positivity within each diagnosis grade for 3 age groups (<30, 30–39, > = 40). We examined bivariate associations between single and multiple infections and 1) disease grade, and 2) age group using Pearson's χ^2^ test. Two-sided statistical tests were considered significant at the alpha level of 0.05. Data were analyzed using SAS (v 9.1, SAS Institute, Cary, NC, 2002).

## Results

Of 299 specimens submitted, 17 were not processed because the block did not have diagnostic material (5.7%). Of the 282 samples processed for HPV testing, 15 (5.3%) were inadequate by LA only, 7 (2.5%) by LiPA, and 4 (1.4%) by both methods (*p*<.01). One sample was only tested with LA due to insufficient residual volume for LiPA assay. The remaining 255 samples with valid results in LA and LiPA were used for comparison of the methods. When comparing LA to unequivocal LiPA results for 25 types common to both assays, individual type concordance ranged from 0.33 to 1.00 (median 0.76) (Table 1). Concordance for detecting any 14 HR type was 0.61 and concordance for detecting any LR type was 0.20; concordance was better when analysis was restricted to the 4 LR types common to both assays (kappa = 0.53 and 0.65, respectively). In all cases, agreement between LA and LiPA was worse when possibly present LiPA results were interpreted as positive. Concordance declined the most for types 39, 52, and 54, to 0.30, 0.31, and 0.05, respectively. In all subsequent analyses, “possible type” LiPA results were considered negative. McNemar's test detected statistically significant differences in marginal proportions for HPV 16, 51, 52, and any HR-HPV. Discordant results were more often LiPA-positive, LA-negative than LA-positive, LiPA-negative.

**Table 1:** Concordance between Linear Array (LA) and LiPA test results for HPV types included in both assays.

HPV TYPE	LA+ LiPA+	LA+ LiPA−	LA− LiPA +	LA− LiPA−	*kappa*	*P* (McNemar's)
**6**	1	1	1	252	0.50	1.00
**11**	1	0	2	252	0.50	0.16
**26**	1	0	0	254	1.00	–
**40**	1	0	0	254	1.00	–
**53**	1	0	2	252	0.50	0.16
**54**	0	1	0	254	–	–
**69**	0	1	0	254	–	–
**70**	2	0	0	253	1.00	–
**71**	0	0	0	255	–	–
**73**	1	3	0	251	0.40	0.08
**82**	2	0	3	250	0.57	0.08
**16**	123	3	15	114	0.86	<.01
**18**	17	2	2	234	0.89	1.00
**31**	13	1	6	235	0.77	0.06
**33**	12	2	5	236	0.76	0.26
**35**	6	1	2	246	0.79	0.56
**39**	4	1	2	248	0.72	0.56
**45**	8	4	1	242	0.75	0.18
**51**	8	0	11	236	0.57	<.001
**52**	16	4	16	219	0.57	<.01
**56**	5	1	1	248	0.83	1.00
**58**	12	2	1	240	0.88	0.56
**59**	1	4	0	250	0.33	0.05
**66**	5	0	4	246	0.71	0.05
**68**	2	0	0	253	1.00	–
**Any high risk (HR)**	225	0	16	4	0.61	<.0001

*Note.* High risk HPV types include 16, 18, 31, 33, 35, 39, 45, 51, 52, 56, 58, 59, 66, and 68. Restricted to samples with adequate genotyping results for both methods (N = 255). Kappa was calculated only on samples that were positive on both tests.

### Algorithm testing

As expected, the parallel algorithm yielded the highest prevalence of any HPV type (95.7%) as well as HR HPV (93.1%) (Table 2). Applying the serial algorithm resulted in nearly the same HR-HPV prevalence as the parallel algorithm (92.1%). However, the prevalence of a few types appeared somewhat lower by application of the serial algorithm, particularly HPV 16 (from 52.7% to 50.2%), HPV 33 (from 7.6% to 5.8%), and HPV 52 (from 13.4% to 8.3%). The median number of HR types per woman (one) did not differ by testing algorithm. The mean number of types per woman was slightly higher using the parallel algorithm (1.31) than for the serial (1.14). Based on the similarity of results using the parallel and serial algorithms, and the efficiency of only performing two tests in a subset of samples in future genotyping projects, we report the remainder of our results according to the serial testing algorithm. Of the 278 valid results, 248 (89.2%) are from LA.

**Table 2:** Prevalence of HPV high risk and low risk types, depending on algorithm for combining linear array (LA) and LiPA results.

			HPV Type Prevalence According to Testing Algorithm			
HPV Type	Included in LA	Included in LiPA	Parallel		Serial	
			N	%	N*	%*
**6**	X	X	3	1.1	3	1.1
**11**	X	X	3	1.1	1	0.4
**16**	X	X	146	52.7	139	50.2
**18**	X	X	22	7.9	20	7.2
**26**	X	X	1	0.4	1	0.4
**31**	X	X	24	8.7	21	7.6
**33**	X	X	21	7.6	16	5.8
**35**	X	X	10	3.6	9	3.3
**39**	X	X	8	2.9	7	2.5
**40**	X	X	1	0.4	1	0.4
**42**	X		3	1.1	3	1.1
**43**		X	0	0	0	0
**44**		X	1	0.4	0	0
**45**	X	X	16	5.8	15	5.4
**51**	X	X	19	6.9	13	4.7
**52**	X	X	37	13.4	23	8.3
**53**	X	X	3	1.1	2	0.7
**54**	X	X	1	0.4	1	0.4
**55**	X		0	0	0	0
**56**	X	X	9	3.3	8	2.9
**58**	X	X	15	5.4	14	5.1
**59**	X	X	5	1.8	5	1.8
**61**	X		0	0	0	0
**62**	X		1	0.4	1	0.4
**64**	X		0	0	0	0
**66**	X	X	9	3.3	5	1.8
**67**	X		1	0.4	1	0.4
**68**	X	X	2	0.7	2	0.7
**70**	X	X	2	0.7	2	0.7
**71**	X	X	0	0	0	0
**72**	X		0	0	0	0
**73**	X	X	5	1.8	5	1.8
**74**		X	1	0.4	0	0
**81**	X		0	0	0	0
**82**	X	X	6	2.2	4	1.4
**83**	X		1	0.4	1	0.4
**84**	X		1	0.4	1	0.4
**89**	X		2	0.7	2	0.7
**IS39**	X		2	0.7	2	0.7
**Any High Risk** ‡	X	X	258	93.1	255	92.1
**Any HPV**	X	X	265	95.7	265	95.7

* LiPA performed if LA was inadequate or if LA was negative for any type; positive result = positive by LA unless LA was inadequate or negative for any type, otherwise positive result = positive by LiPA (N = 277).

‡ Includes HPV types 16, 18, 31, 33, 35, 39, 45, 51, 52, 56, 58, 59, 66, 68.

Of the 278 specimens with typing results, 32.7% (n = 91) were CIN2, 31.3% (n = 89) were CIN3, and 30.9% (n = 86) were SCC. AIS comprised <1% of specimens (n = 2), and ADC accounted for only 2.5% (n = 7). The remaining 5 specimens were from subjects who were diagnosed with cervical cancer other than SCC and ADC (classified as ‘other invasive’), for a total of 98 (35.3%) invasive lesions. Proportionally higher CIN2 lesions (14.3%) were typed by LiPA than CIN3/AIS (12.4%) or invasive lesions (6.1%), but the difference was not statistically significant (p = .17).

Among women with race information (n = 204) in all diagnosis grades, about half were non-Hispanic white, a third were non-Hispanic black, and 10% were Hispanic. Median age increased with increasing severity of squamous lesions, from 27 years in CIN2 to 33 in CIN3 to 40 in ICC (p<.0001). Median age for AIS was higher at 41 years and highest for ADC (48 years).

### Type prevalence

Table 3 presents HPV DNA prevalence by diagnosis category. No HPV DNA was detected in 11 specimens, 9 of which were from invasive cancers. HR HPV was detected in 96.6% of CIN2, 95.5% of CIN3/AIS specimens and 85.7% of specimens with invasive cancer (data not shown). HPV type 16 was the most common in all diagnosis categories (except 7 ADC) and increased from 45.6% in CIN2 to 52.8% in CIN3/AIS to 56.3% in SCC specimens.

**Table 3:** HPV type prevalence among females diagnosed with CIN2+, North Carolina, 2001–2006.

	CIN2N = 91		CIN3/AISN = 89		SCCN = 86		ADCN = 7		Other InvasiveN = 5		TotalN = 278	
	n	(%)	n	(%)	n	(%)	n	(%)	n	(%)	n	(%)
**Negative**	1	(1.1)	1	(1.1)	5	(5.7)	3	(42.9)	1	(20.0)	11	(4.0)
**HR HPV**												
**16**	41	(45.6)	47	(52.8)	49	(56.3)	1	(14.3)	2	(40.0)	140	(50.4)
**18**	7	(7.8)	5	(5.7)	6	(6.9)	1	(14.3)	1	(20.0)	20	(7.2)
**31**	10	(11.1)	9	(10.3)	1	(1.1)	–	–	–	–	20	(7.2)
**33**	4	(4.4)	7	(8)	4	(4.6)	1	(14.3)	–	–	16	(5.8)
**35**	2	(2.2)	3	(3.4)	3	(3.4)	–	–	1	(20.0)	9	(3.2)
**39**	5	(5.6)	2	(2.3)	–	–	–	–	–	–	7	(2.5)
**45**	2	(2.2)	4	(4.6)	8	(9.2)	1	(14.3)	–	–	15	(5.4)
**51**	9	(10)	3	(3.4)	1	(1.1)	–	–	–	–	13	(4.7)
**52**	12	(13.3)	8	(9.2)	3	(3.4)	–	–	–	–	23	(8.3)
**56**	4	(4.4)	–	–	4	(4.6)	–	–	–	–	8	(2.9)
**58**	6	(6.7)	7	(8)	1	(1.1)	–	–	–	–	14	(5)
**59**	5	(5.6)	–	–	–	–	–	–	–	–	5	(1.8)
**66**	4	(4.4)	–	–	1	(1.1)	–	–	–	–	5	(1.8)
**68**	1	(1.1)	1	(1.1)	–	–	–	–	–	–	2	(0.7)
**Possible HR**												
**26**	1	(1.1)	–	–	–	–	–	–	–	–	1	(0.4)
**53**	1	(1.1)	1	(1.1)	–	–	–	–	–	–	2	(0.7)
**67**	–	–	1	(1.1)	–	–	–	–	–	–	1	(0.4)
**69**	–	–	–	–	1	(1.1)	–	–	–	–	1	(0.4)
**73**	3	(3.3)	–	–	2	(2.3)	–	–	–	–	5	(1.8)
**82**	1	(1.1)	–	–	1	(1.1)	–	–	–	–	2	(0.7)
**HPVX**	2	(2.2)	–	–	–	–	–	–	–	–	2	(2.2)
**IS39**	–	–	2	(2.3)	–	–	–	–	–	–	2	(0.7)
**LR HPV**												
**6**	3	(3.3)	–	–	–	–	–	–	–	–	3	(1.1)
**11**	1	(1.1)	–	–	–	–	–	–	–	–	1	(0.4)

CIN2+: cervical intraepithelial neoplasia grades 2/3 and adenocarcinoma in situ (AIS).

*Note*. 2 AIS cases with single HPV 16 infection are combined with CIN3.

**Figure 1:**
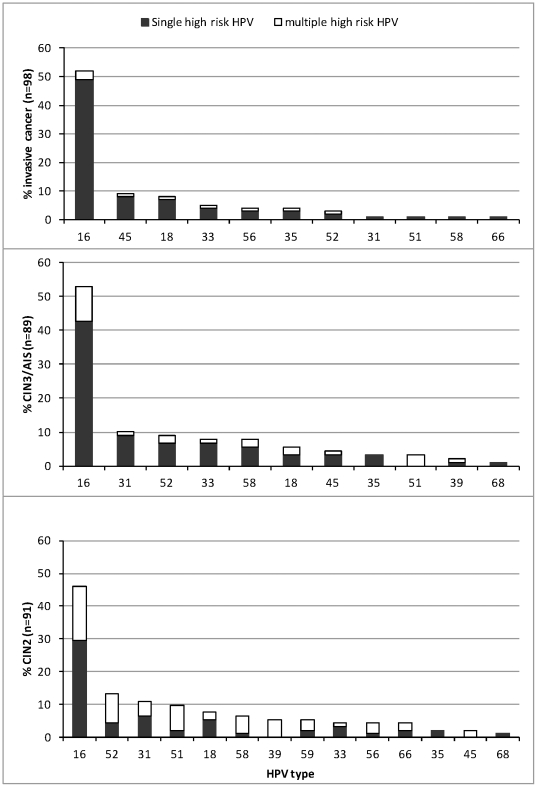
Distribution of single and multiple high risk (HR) HPV types by diagnosis grade.

Vaccine type HPV 16 or 18 was detected in 59.2% of invasive cancer specimens. As shown in Figure 1, HPV 45 was the second most common type among invasive cases (9.2%), slightly more than HPV 18 at 8.2%. Prevalence of other phylogenetically related HPV types detected among invasive cases was 5.4% for HPV 33, 4.1% for HPV 35, 3.1% for HPV 52 and 1.0% for HPV 58. LR HPV types 6, 11, 40, 42, 54, 83, 84, and 89 were detected in approximately 10% of CIN2 lesions, mostly as a co-infection with HR HPV types except in two CIN2 (HPV 6 and 42) and one CIN3 (HPV 62) specimens (data not shown). No LR HPV types were detected in SCC, ADC, or other invasive cervical specimens.

Overall, HPV 16 or 18 was detected in 56.5% of specimens. The proportion of HPV 16/18-related lesions appeared to increase with disease severity, although this trend was not statistically significant (Table 4). We found no statistical difference in the proportion of 16/18-related lesions by race or by age category, overall or stratified by disease severity as shown in Table 4.

**Table 4:** HPV 16 and 18 type prevalence in cervical intraepithelial lesions, by diagnosis and demographics.

	Overall HPV 16/18-related lesions		HPV 16/18-related CIN2 lesions		HPV 16/18-related CIN3/AIS lesions		HPV 16/18-related invasive lesions	
	Number of women	%	Number of women	%	Number of women	%	Number of women	%
**Overall**	278	56.5	91	52.8	89	57.3	98	59.2
**Race/ethnicity**								
NH white	94	57.5	26	46.2	34	61.8	33	63.6
NH black	71	54.9	26	53.9	23	47.8	22	63.6
Hispanic	25	40.0	9	33.3	10	40.0	6	50.0
Other	14	78.6	6	50.0	5	100.0	3	100.0
*P*	*0.13*		*0.79*		*0.11*		*0.53*	
**Age, years**								
<30	90	54.4	57	52.6	30	56.7	3	66.7
30–39	90	57.8	27	55.6	44	54.6	19	66.7
> = 40	98	57.1	7	42.9	15	66.7	76	63.9
*P_trend_*	*0.77*		*0.87*		*0.90*		*0.84*	

NH: non-Hispanic.

CIN: cervical intraepithelial neoplasia.

Figure 1 displays HPV type distribution by single and multiple type infection. Among SCC and ADC cases, 94.4% had only a single HR HPV detected; single infections were less common in CIN3 and AIS cases (86.4%) and lowest in CIN2 specimens (65.6%) (p<.0001). Multiple type infection decreased with increasing age group (58% in females <30, 27.7% in females aged 31–49, and 14.9% in females > = 50 years; p<.0001).

## Discussion

Prevalence and type distribution of HPV in cervical precancers and invasive cancers is not well-described in the United States [11]. Using archived FFPE biopsy tissue from a large source population of females with histologically-confirmed precancerous and invasive cervical lesions, we examined HPV type prevalence and evaluated laboratory methods for HPV DNA detection in FFPE biopsy tissue. Specifically, we used a combination of LA and LiPA HPV genotyping assays to maximize HPV DNA detection in archived biopsy specimens.

Several studies have reported high concordance between the LA and LiPA assays for detecting HPV DNA in cervical cells [10], [12], [13], [14]. However, assay performance on cytology specimens are not directly comparable to that for FFPE specimens given the potential for DNA degradation in the latter. Fewer studies have investigated the use of commercial assays for HPV genotyping in FFPE specimens. In a study from Australia, Tan et al. compared use of LA vs LiPA for HPV genotyping of a small sample of vulval biopsy specimens, and concluded that LiPA has higher sensitivity to detect DNA in archived biopsy tissue [15]. A larger study by Wheeler et al. conducted in a U.S. population, however, found high (>95%) crude agreement between the 2 assays for most HPV DNA types in cervical FFPE tissue [6]. Consistent with the U.S. study, our data indicate that using a serial testing algorithm whereby only HPV-negative and inadequate samples by LA are tested with LiPA results in very little change in prevalence estimates of HR-HPV for most types compared to testing all specimens with both tests in parallel. Using a serial algorithm, we demonstrate greatly improved efficiency without compromise to sensitivity in detection or bias in detection of HR HPV types. If only LA negative or inadequate samples had been tested by LiPA, 238 tests could have been avoided.

As expected, HPV16 was the most commonly detected HPV type in all diagnosis categories, followed by HPV 52, 18, 31, 33, and 45. These findings are similar to that found in non-Hispanic white and Hispanic women with CIN3/AIS and invasive cervical cancer in the U.S. [6], as well as in other international settings [11], [16]. We found that HPV16 was more likely to occur as a single infection in CIN3/AIS and invasive cancers compared to CIN2. This could be due to differential clearance of HR HPV types, but the results may be confounded by age which we were unable to control for given the small sample size of the study [17], [18], [19]. HPV16 and 18 were less commonly detected in CIN2 and CIN3/AIS than ICC lesions, and distribution of HR types differed by lesion grade. Among invasive cases, HPV45 was the second most common type (10%); HPV18 was third highest (8%) followed by 33 (6%). We found less HR HPV positivity in invasive cases compared to lower grade lesions. The reasons for this are unclear and could reflect differential specimen quality. However, the proportion of invasive cases associated with HR HPV (85.7%, 95% CI: 78.8–92.6) is similar to that found in other studies. For example, Wheeler et al. reported a 90.0% HPV positivity in U.S. women with invasive cervical cancer compared to 97.1% HPV positivity in those with CIN3 or AIS [6].

Our study has several limitations. First, our population was restricted to adult female residents of North Carolina and may not be generalizable to other U.S. populations. However, we obtained specimens from 4 large pathology laboratories that serve a large majority of residents of North Carolina to minimize selection bias. Second, because the study was cross-sectional in nature, it was neither possible to investigate the effects of aging on HPV persistence or type distribution, nor to examine the influence of HPV types on disease progression. Third, we had limited statistical power to investigate HPV coinfection patterns by disease grade. Fourth, we did not perform additional testing to validate typing results discrepant between LA and LiPA. We assumed that results positive by either method were correct, but some might actually be falsely positive. Concordance between LA and LiPA may differ depending on method of sample collection, extraction method, or disease prevalence.

In summary, we used specimens available from a pilot study to develop an efficient approach to HPV testing of archived FFPE specimens. Currently, the serial algorithm is being applied to a variety of other population-based studies of HPV type distribution in cancer and precancer specimens (CDC, unpublished data) [6]. Our data also provide important information about the distribution of individual HPV types in cervical disease prior to HPV vaccine introduction in North Carolina. The impact of HPV vaccines in reducing the burden of cervical and other HPV-associated cancers is of greatest concern, but it may take many years to demonstrate population-level reductions in these diseases. Cervical precancer lesions (CIN2/3) take less time to develop and are the accepted proxy for monitoring the impact of vaccine on cervical cancer. North Carolina has a large rural population at higher risk of cervical cancer, and an annual cervical cancer incidence of 8.2 cases/100,000 women [20], [21]. Thus, these baseline results may be a useful contribution to monitoring primary and secondary cervical cancer prevention efforts in North Carolina.
